# Cross-modal size-contrast illusion: Acoustic increases in intensity and bandwidth modulate haptic representation of object size

**DOI:** 10.1038/s41598-019-50912-8

**Published:** 2019-10-08

**Authors:** Maiko Uesaki, Hiroshi Ashida, Akiyoshi Kitaoka, Achille Pasqualotto

**Affiliations:** 1 School of Social Sciences, Nanyang Technological University, Singapore, Singapore; 2 Open Innovation & Collaboration Research Organization, Ritsumeikan University, Osaka, Japan; 3 Japan Society for the Promotion of Science, Tokyo, Japan; 4 Graduate School of Letters, Kyoto University, Kyoto, Japan; 5 College of Letters, Ritsumeikan University, Kyoto, Japan; 6 Faculty of Arts and Social Sciences, Sabanci University, Istanbul, Turkey; 7 School of Psychology, Faculty of Science and Engineering, University of Nottingham Malaysia, Selangor Darul Ehsan, Malaysia

**Keywords:** Sensory processing, Sensory processing, Human behaviour, Human behaviour

## Abstract

Changes in the retinal size of stationary objects provide a cue to the observer’s motion in the environment: Increases indicate the observer’s forward motion, and decreases backward motion. In this study, a series of images each comprising a pair of pine-tree figures were translated into auditory modality using sensory substitution software. Resulting auditory stimuli were presented in an ascending sequence (i.e. increasing in intensity and bandwidth compatible with forward motion), descending sequence (i.e. decreasing in intensity and bandwidth compatible with backward motion), or in a scrambled order. During the presentation of stimuli, blindfolded participants estimated the lengths of wooden sticks by haptics. Results showed that those exposed to the stimuli compatible with forward motion underestimated the lengths of the sticks. This consistent underestimation may share some aspects with visual size-contrast effects such as the Ebbinghaus illusion. In contrast, participants in the other two conditions did not show such magnitude of error in size estimation; which is consistent with the “adaptive perceptual bias” towards acoustic increases in intensity and bandwidth. In sum, we report a novel cross-modal size-contrast illusion, which reveals that auditory motion cues compatible with listeners’ forward motion modulate haptic representations of object size.

## Introduction

Being able to accurately estimate object size is essential in guiding our interaction with the environment. However, the perceptual representations of object size are not inherently determined by one source of information; rather, they are constructed by integrating multiple sources of information.

For example, within vision, the size of an object is computed based not only on its projected retinal image size, but also on other sources of information provided by the environment. Much of the evidence for the roles different sources of information play in the estimation of object size comes from sensory illusions whereby the size of an object is misperceived. One of such illusions is the Ebbinghaus illusion^1,2^. In the Ebbinghaus illusion, the central target object appears smaller when surrounded by an array of similar objects that are larger, as compared to when surrounded by an array of the similar objects of the identical size. The Ebbinghaus illusion demonstrates how the surrounding context can influence the visually perceived size of the target object.

The information potentially relevant to the determination of object size can also be generated by multiple modalities. Evidence is accumulating of the interaction of multiple sensory modalities, in construction of the perceptual representations of object size. The effect of looming sounds on visual perception of object size, for example, illustrates how the context provided by auditory information influences the visually perceived size of objects. It has been shown that being exposed to auditory information indicative of approaching sound sources (i.e. looming sounds) leads to visually perceived increases in the size of the target object^3–6^. Not only does the effect of looming sounds on visual perception of object size indicate that inputs from multiple modalities can be integrated in order for perceptual representations of object size to be constructed, but it also demonstrates the prominence of sensory motion cues.

Sensory information indicative of approaching objects, either due to the observer moving towards the objects or to the objects moving towards the observer, provide critical information for our navigation through the environment. One important sensory cue to the change in distance between the observer and the objects in the environment derives from the observer’s self-motion, and is primarily represented in the retinal size of the objects. For example, an increase in the retinal size of trees on the sides of a road would indicate the observer’s forward motion along the road. Although the dominant cue to self-motion is the pattern of retinal motion resulting from the observer’s own motion (i.e. optic flow^7,8^) under typical circumstances, this moving pattern is often confounded by eye movements^9–12^ and motion of other objects in the visual field^8,13,14^, which render sensory information from other modalities such as audition highly relevant. Auditory cues to self-motion tend to be less compelling compared to those of the visual modality, however, characteristics of auditory modality such as high temporal sensitivity and acuity are instrumental in the evaluation of self-motion, and the resulting change in distance between the moving individual and the stationary objects in the environment^15^.

Auditory cues to self-motion are even more critical in visually impaired individuals deprived of visual cues to self-motion. In fact, sensory substitution tools have been developed to convert images into forms that the visually impaired can process with sensory modalities other than vision, in order to assist the visually impaired in using visual information as supplement to other sensory cues in guiding their navigation. One of the sensory substitution devices that transforms visual information to auditory information is software called “the vOICe.” The vOICe scans visual images from left to right, converts them into greyscale images, and subdivides them into pixels; each of which is then converted to a sound based on its position within the scanned image, and its luminance. The horizontal positions of the pixels are expressed in terms of order (i.e. sounds representing pixels on the left are played before those representing pixels to their right), the vertical positions in terms of pitch (i.e. sounds representing pixels towards the top of the image have a higher pitch than those towards the bottom), and the luminance in terms of loudness (i.e. sounds representing pixels of higher luminance are louder than those of lower luminance). Although originally developed to assist the visually impaired, sensory substitution tools have since been used in research conducted with both visually impaired as well as sighted individuals^16–18^. In this study, the snapshots of visual input during linear motion along a tree-lined road were converted into their auditory equivalents, using the vOICe, in order to investigate what impact auditory cues to self-motion may have on perception of object size.

Evidence from the effect of looming sounds on the perceived size of objects suggests that auditory cues increasing in intensity and bandwidth, compatible with forward self-motion, would lead to overestimation of the size of an object. Sutherland and colleagues^19^ explain that when sounds increasing in intensity are associated with static visual objects they bias the visual perception of the size of those objects, as in-depth auditory motion cues are re-mapped onto visual features. Furthermore, it has also been shown that visual perception of size can be modulated by auditory cues to distance^20^. It is interesting to note, however, that Serino *et al*.^21^ reported differential responses to stimuli that are further away and to those that are close enough to interact with the body. This raises the question: Do auditory motion cues modulate the construction of perceptual representations of object size, only when the auditory cues are re-mapped onto the *target* object (i.e. the object, of which size is to be estimated) as to the looming sound effect, or also when the sounds are re-mapped onto the *environment* (i.e. the context) in which the listener and the target object are placed within the interactable distance? And if so, how?

In this article, we report on a novel cross-modal size-contrast illusion that demonstrates the contribution of auditory motion cues in the modulation of haptic representation of object size.

## Results

The aim of this experiment was to determine how haptic size perception is affected by the exposure to auditory information compatible with forward motion, backward motion, and incompatible with any linear motion. Results reported below are based on the performance of all 66 participants. Blindfolded participants were asked to estimate the lengths of three wooden sticks through haptic exploration while exposed to the auditory stimulus compatible with either their forward motion (Forward Condition) or backward motion (Backward Condition) along a tree-lined path, or the auditory stimulus without any motion components (Scrambled Condition).

Before analysing the difference between the three conditions, we determined whether there was any effect of the order in which the sticks of three different lengths were presented. A three-by-three factorial ANOVA with Condition (Forward vs. Backward vs. Scrambled) and Order (first vs. second vs. third) as factors showed no significant main effect of Order (F (2, 126) = 0.27, p = 0.76), and no significant interaction between Condition and Order (F (4, 126) = 0.41, p = 0.80).

We also confirmed that there were no significant differences between male and female participants in any of the three conditions with a three-by-two factorial ANOVA with Condition and Sex (male vs. female) as factors (F (1, 60) = 0.16, p = 0.69), and that there was no interaction between Condition and Sex (F (2, 126) = 0.40, p = 0.68). Although sex differences have been reported in the literature of looming sounds and their influence on perception^22,23^, since we found no differences between sexes, this variable will not be discussed.

Table 1 describes the average length estimates for the three wooden sticks and standard errors (SEs) of the means for each condition. For Forward Condition, the lengths of all three objects were consistently underestimated. In contrast, for Backward Condition and Scrambled Condition, the direction of error (i.e. over- or under-estimation) was not consistent across the three lengths.

**Table 1:** Average length estimates for the 15-, 25-, 40-cm long sticks and standard errors (SEs) of the means reported in brackets (n = 22 per condition).

	15 cm	25 cm	40 cm
*ForwardCondition*	11.73 cm (0.86)	21.14 cm (1.01)	33.14 cm (1.35)
*BackwardCondition*	14.56 cm (0.85)	25.47 cm (1.10)	41.23 cm (1.76)
*ScrambledCondition*	13.68 cm (0.98)	25.1 cm (1.71)	39.1 cm (2.17)

As Table 1 shows, there was a consistent margin of error in the length estimations regardless of the conditions; however, the errors were notably larger for all lengths for Forward Condition than for Backward Condition and Scrambled Condition. The percentage of error for the 15-, 25-, 40-cm lengths was 22%, 15% and 17%, respectively, for Forward Condition, as compared with 3%, 2% and 3% for Backward Condition and 9%, 0.3% and 2% for Scrambled Condition.

**Figure 1:**
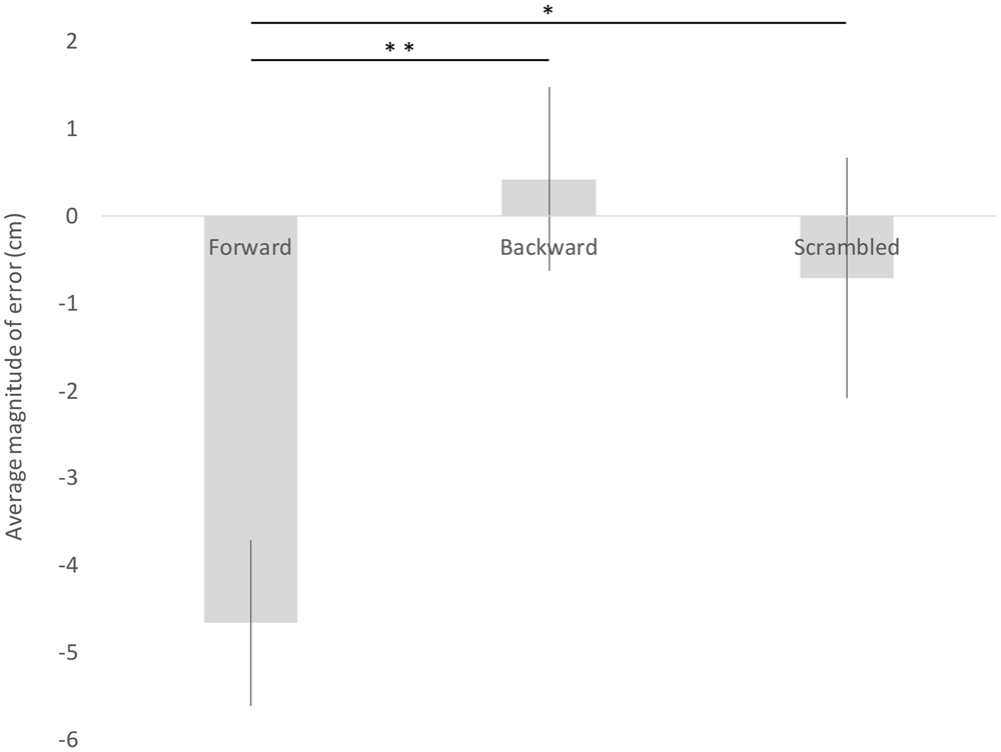
Magnitude of size-estimation error in centimetres for Forward Condition, Backward Condition and for Scrambled Condition (* significant at p < 0.05; ** significant at p < 0.01). Error bars indicate +/−1 SE.

In Fig. 1, the magnitude of error in size estimation averaged across the three objects is plotted for each auditory condition. The average magnitude of error was 4.67 cm (SE = 0.95) for Forward Condition; 0.42 cm (SE = 1.05) for Backward Condition; and 0.71 cm (SE = 1.38) for Scrambled Condition. There was a statistically significant difference between conditions as determined by one-way ANOVA (with Condition as a factor; F (2, 63) = 5.47, p < 0.01). Subsequent Tukey’s honestly significant difference (HSD) post hoc test showed that Forward Condition differed significantly from Backward Condition at p < 0.01 and Scrambled Condition at p < 0.05. There was no significant difference between Backward Condition and Scrambled Condition (p = 0.74).

Length estimates in Forward Condition and Backward Condition were also analysed with regards to Scrambled Condition (as opposed to the actual lengths of the objects) as a baseline. Two-tailed one-sample t-test revealed that the difference between Forward Condition and Scrambled Condition was significantly larger than 0 (t (65) = −3.37, p < 0.01, two-tailed), despite the small error observed in Scrambled Condition in the same direction as in Forward Condition (Fig. 1). This provides further evidence for the extent of underestimation in Forward Condition. Contrarily, the magnitude of error in Backward Condition compared with that in Scrambled Condition was not significantly different from 0 (t (65) = 0.86, p = 0.40, two-tailed). In line with the results of analyses whereby the actual lengths of the objects were considered a baseline, an independent-samples t-test yielded that the magnitude of error in size estimation differed significantly between Forward Condition and Backward Condition (t (130) = −2.88, p < 0.01, two-tailed) when the estimates in Scrambled Condition were used as a baseline.

## Discussion

This study investigated the effect of auditory cues to self-motion on the construction of haptic representations of object size, and we discovered a novel cross-modal size-contrast illusion that demonstrates the contribution of auditory motion cues in the modulation of haptic representations of object size.

Results showed that the lengths of the objects were consistently underestimated during the exposure to auditory motion cues increasing in intensity and bandwidth, and therefore compatible with forward self-motion. This phenomenon resembles the dynamic Ebbinghaus illusion, which was recently reported by Mruczek and colleagues^24,25^. They modified the classic Ebbinghaus illusion so that the target object was surrounded by an array of similar objects that expanded and contracted in size and bandwidth. In the dynamic Ebbinghaus illusion, the concept of motion is introduced as another factor that can influence the construction of the perceived size of the target object. Mruczek and colleagues^24,25^ showed that the target object was perceived to be smaller as the surrounding objects increased in size, much like what was observed in our experiments. The dynamic Ebbinghaus illusion^24,25^ reveals the prominent role of motion information in determining visual representations of object size. Our results suggest that the mechanism underlying the dynamic Ebbinghaus illusion may be present across modalities. By contrast, our results may seem contradictory to the evidence from the looming sound effect, which is defined by the perceived increase in the size of the target object due to the acoustic increase in intensity^3–6^. However, the different effects of auditory motion cues on the perceived size of the target object observed in the case of the looming sound effect and in our experiment can be accounted for by the objects to which the auditory cues are assigned. In the case of the looming sound effect, the auditory cues are re-mapped onto the *target* object itself, whereas in our study, the association was established between the sound and the object that constituted the *environment* (i.e. the context) within which the listener and target object were placed, prior to the experiment.

The findings indicate that the phenomenon observed here is a novel cross-modal size-contrast illusion, that reveals that auditory motion cues compatible with forward motion contribute to modulation of the perceived size of objects. As mentioned above, this illusion may share some aspects with the dynamic Ebbinghaus illusion^25^, whilst it is unique in other aspects.

One of the most intriguing aspects shared between the cross-modal size-contrast illusion observed here and the dynamic Ebbinghaus illusion, is the motion component in the context in which the target object is placed. The auditory stimuli used in this experiment were generated by converting visual images simulating snapshots during linear motion along a tree-lined road into their auditory counterparts. Therefore, acoustic increases and decreases in intensity and bandwidth were compatible with the listener’s forward and backward motion, respectively. Such auditory motion cues to self-motion through the environment over time, are indicative of the listener’s shift in both time and space. As explained by a theory of magnitude (ATOM^26^), connections between conceptual elements such as time and quantity are a well-established phenomenon^27^, and are also echoed in other parallels such as space in quantity^28^. There is also neurological evidence linking time, space, and size perception^29^, derived from observations of those with parietal cortex lesion. Based on previous findings^30–34^, one of the functions of the parietal cortex is to encode information regarding magnitudes in the environment (e.g. how far, how fast, how long), with respect to action (i.e. motor planning). ATOM may, at least in part, explain the mechanism underlying the size-contrast illusion described here. As the auditory stimuli used in this experiment were compatible with the listener’s linear motion along a path, they were indicative of the speed of motion, the distance travelled, and how close the stationary objects in the environment were to the listener; and those auditory cues were processed with respect to the task that involved proprioceptive perception and judgement (i.e. estimation of object size). The illusion highlights how important sensory cues to self-motion are, as they enable us to estimate the direction and velocity of our movement through the environment, to recognise our position relative to the objects in the environment, to calculate the time-to-contact with those objects, and to conduct motor planning^35,36^.

On the other hand, certain aspects of the illusion observed here differ from the Ebbinghaus illusion. The most fundamental of those is the fact that one is cross-modal whilst the other is uni-modal. Most of size-contrast illusions can be described as sources of information from one modality (e.g. vision in the case of the Ebbinghaus illusion) contributing to the modulation of perceptual representations of object size within the same modality. Contrarily, the illusion observed here is the result of the effect of acoustic changes in intensity and bandwidth on haptic representations of object size. However, it should be noted that, in this case, it may not be as straightforward as the auditory input modulating haptic perception. This is due to the application of sensory substitution in the rendering of auditory stimuli in our study, and the pre-experimental training during which “calibration” of the auditory stimuli and their visual origins took part. As demonstrated in Pasqualotto and Esenkaya^37^, visual stimuli can be re-mapped onto auditory dimension through training (see also^17,38^). Although participants were blindfolded during the experiment, they had learnt beforehand the association between the visual and auditory representations of the object that constituted the surrounding in which the target object was placed (i.e. the pine tree). According to Nanay^39^ (see also^40^), sensory substitution assisted perception (e.g. vision) may not be the same as vision as typically defined, or perception through the substituting sensory modality (e.g. audition); but instead, it may be multimodal mental imagery^41,42^. This is corroborated by a body of research that has shown that activity in the visual cortex can be elicited in the blind or blindfolded, by corresponding sensory stimulation in other sensory modalities (for examples, see^43–45^). The notion that the cross-modal size-contrast illusion observed here was induced by visual mental imagery triggered by auditory sensory stimulation, cannot be rejected until further investigated.

Results also showed that the perceived lengths of the same objects were little influenced by the auditory motion cues decreasing in intensity and bandwidth, compatible with backward self-motion. In fact, estimation of lengths was as accurate during the exposure to sounds compatible with backward motion as during the exposure to the same set of sounds presented in a scrambled order (i.e. auditory stimuli with no motion component). This may seem counter-intuitive as it might be expected that acoustic decreases in intensity and bandwidth would have the opposite effect to acoustic increases in intensity and bandwidth, and thus lead to overestimation of object size. Although the phenomenon observed is unidirectional; i.e. the effect was only present when subjects experienced the auditory stimulation compatible with forward motion, but not when they experienced auditory stimulation compatible with backward motion, it would be premature to discard the effect completely considering how consistent and robust the effect was during auditory stimulation with increasing intensity and bandwidth. It is possible that the illusion only occurs during exposure to auditory stimulation compatible with forward motion, because acoustic increases in intensity and bandwidth tend to be more relevant and salient compared to decreases in intensity and bandwidth, because they are often indicative of objects moving further from the listener^6,46–48^. For example, an approaching car is potentially more threatening than a car that is moving away. Likewise, stationary objects towards which the observer is moving should be more relevant to the observer’s safety and navigation than objects from which they are moving away, since the former would require the observer to take appropriate measures in order to avoid colliding with them. This is described in the “adaptive perceptual bias” reported by Neuhoff ^6^, which suggests that increases of acoustic intensity are perceptually salient as they can indicate an approaching sound source in the environment, which can potentially be threatening. This bias towards approaching sounds provides a temporal margin of safety^48^, by allowing for the time necessary for defensive motor action (e.g. avoidance). The different effects auditory motion cues of increasing and decreasing intensity and bandwidth had on the construction of haptic representations of object size are also in line with previous findings that sounds of increasing intensity and bandwidth are processed differently from sounds of decreasing intensity and bandwidth in the auditory domain^4,5,49,50^, and that sounds of increasing intensity and bandwidth elicit greater physiological responses (i.e. skin conductance and phasic alertness) compared to sounds of decreasing intensity and bandwidth^3^.

Our findings introduce a novel cross-modal illusion of relative size perception. It illustrates that exposure to acoustic increases in intensity and bandwidth compatible with forward motion lead to underestimation of the size of objects by touch. The fact that error of such magnitude was not induced by acoustic decreases in intensity and bandwidth compatible with backward motion suggests that “adaptive perceptual bias”^6^ may influence the illusory effect. In order to determine whether it is indeed the potential threat posed by the sources of acoustic increases in intensity and bandwidth that lead to the underestimation of object size, future study should systematically manipulate the level of threat posed by the sound sources and assess whether the level of threat is reflected in the magnitude of error in haptic size estimation (i.e. magnitude of illusory effect). This may be achieved by manipulating components of the motion cues, as to indicate varying distances to, or varying speeds at which the observer is approaching the objects in the environment (e.g. smaller volume indicating greater distance to the objects, ergo less immediate threat; more rapid increases indicating less time-to-contact, ergo more immediate threat). Another aspect of the illusion that this study was unable to clarify due to the confounding nature of the stimuli used, is the role mental imagery plays in the illusion. Future study should address this by comparing those who receive a training to establish the association between the auditory stimuli and the substituted visual stimuli with those who do not receive such a training and therefore are unaware of the original visual images used to render the auditory stimuli. Furthermore, in order to have a more comprehensive understanding of the nature of this new illusion, it is also important to investigate other factors on which the magnitude of this effect depend, such as the number of inducers (i.e. sound sources), and similarities between the target of which size is to be estimated and inducers.

On a more global scale, it would be interesting to see if this illusion generalises to other modalities. For example, whether optic-flow stimulation compatible with forward motion similarly modulates the haptically perceived size of objects.

This illusion opens new avenues to studying cross-modal influences of sensory cues compatible with self-motion, and multisensory interaction, but also has implications in its application for multisensory virtual reality, as well as safety systems that are dependent on sensory perception such as vibrotactile warning signals^51^.

## Materials and Methods

### Participants

Sixty-six healthy volunteers (33 males and 33 females; of the ages between 19 and 23 years) recruited amongst students at Sabanci University participated in the study. Each participant was randomly assigned to one of the three conditions: Forward Condition, Backward Condition, and Scrambled Condition (see Procedure). Mean age (standard deviation; SD) of participants was 21.32 (SD = 1.25), 21.23 (SD = 1.11), 21.23 (SD = 1.51) years for Forward Condition, Backward Condition, and Scrambled Condition, respectively. All had normal or corrected-to-normal vision, and normal hearing. All participants gave written informed consent to take part in this study, which was conducted in accordance with the ethical standards stated in the Declaration of Helsinki and approved by Sabanci University Research Ethics Committee. Participants were compensated for their time with course credits or meal vouchers.

### Auditory stimuli

**Figure 2:**
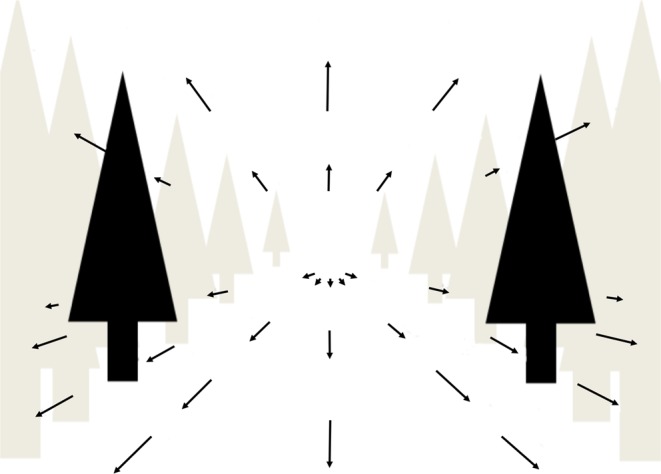
Visual representation of the path with pine trees. Auditory stimuli were either compatible with forward or backward motion along this path, or not compatible with any linear motion.

A series of six images were converted into an auditory format with sensory substitution software called the vOICe (freely available at: www.seeingwithsound.com). Each image represented a visual snapshot during forward motion along a path with a pine tree on each side (Fig. 2).

**Figure 3:**
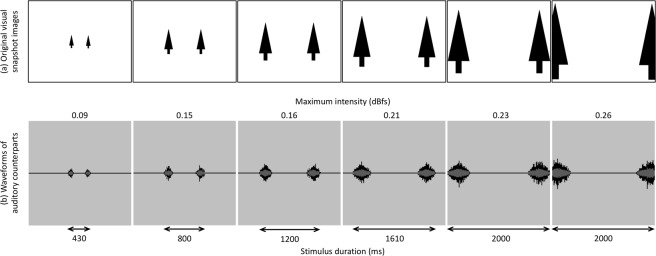
Coupling of (**a**) the original visual images of pine trees and (**b**) waveforms of their auditory counterparts rendered using the vOICe, in the sequence compatible with the observer’s forward motion. The maximum intensity (dBfs) of each auditory stimulus reflected the height of the pine trees as they appear in the corresponding original visual image, and the stimulus duration (ms) the size of the pine trees and the distance between them.

In the first image of the sequence (the leftmost image in Fig. 3a), the pine trees appear small and close to the centre of the screen; in the following images, the pine trees appear bigger and further from the centre of the screen as compared with the preceding image, simulating the observer’s forward motion along the path (Fig. 3a). Figure 3 demonstrates the coupling of the original visual images and the waveforms of their auditory counterparts generated with the vOICe.

Each of the six resulting auditory stimuli represented a pair of pine trees in the auditory modality (Fig. 3b). The duration of each stimulus reflected the size of the pine trees and the distance between them, as they appear in the corresponding original visual image. Stimulus durations were; 430 ms, 800 ms, 1200 ms, 1610 ms, 2000 ms, and 2000 ms. In the last image of the sequence (the rightmost image in Fig. 3a), the pine trees are so far into the peripheral of the “visual field” that a portion of each of them is not “seen”. This is also reflected in its auditory counterpart (the rightmost image in Fig. 3b). The peak loudness intensity of each stimulus represented the height of the pine trees as they appear in the corresponding original visual image. Maximum intensities as expressed in dB full scale (dBfs; ranging from −1 to 1) were: 0.09 dBfs, 0.15 dBfs, 0.16 dBfs, 0.21 dBfs, 0.23 dBfs, and 0.26 dBfs.

Those auditory stimuli were presented using Audacity (www.audacityteam.org) in three different orders, depending on the condition to which the participant was assigned. In one condition, the stimuli were presented in an ascending sequence (i.e. increasing in intensity and bandwidth conveying the pair of pine trees becoming larger and shifting towards the peripheral) compatible with snapshots of the scene during forward motion (Forward Condition); in another, in a descending sequence (i.e. decrease in intensity and bandwidth conveying the trees becoming smaller and shifting towards the centre) compatible with snapshots of the scene during backward motion (Backward Condition). In the control condition, the same set of auditory stimuli was presented in a scrambled order (Scrambled Condition). The total duration of stimulus presentation was 16655 ms in all conditions.

The duration between any two auditory stimuli (i.e. inter-stimulus interval: ISI) was 1400 ms.

### Haptic test material

Three smooth, round-surface wooden sticks (1 cm diameter) of varying lengths (15 cm, 25 cm, and 40 cm) were used as the test objects (i.e. target objects), the lengths of which were estimated with haptics by the participants.

### Procedure

Before the experimental session, participants put on headphones and saw a short PowerPoint presentation on a computer. The presentation briefly introduced the concept of sensory substitution, that sensory information that is normally conveyed through one modality (i.e. vision) is translated into the information compatible with another sensory modality (i.e. audition). It then presented an image of one sample pine tree along with its auditory counterpart that was generated using the vOICe, in order for the participants to learn the coupling between the visual and auditory representations of the pine tree (i.e. calibration). Note that the sample pine tree was identical in its shape as any of the pine trees in the original images used to generate the auditory stimuli for the experiment. The sound of the pine tree was repeated until participants moved onto the next slide of the presentation. Participants were encouraged to look at the image and to listen to the sound for about one minute.

Subsequently, participants were blindfolded (Mindfold™, Colorado, USA) and asked to listen to the auditory stimuli: Those in Forward Condition heard the auditory stimuli presented in an ascending sequence, those in Backward Condition in a descending sequence, and the others in a scrambled order.

After one minute of attending to the auditory stimuli, whilst the sound remained playing, blindfolded participants were handed the three wooden sticks one by one for free haptic exploration using both hands. Each wooden stick was presented once. The presentation order of the three sticks was randomised amongst participants. A verbal estimate (in cm) of the perceived length was made once for each wooden stick by each participant. In total, each participant in each condition generated three estimates. The estimates were reported within a few seconds from the beginning of haptic exploration.

## Supplementary information

Dataset 1

## Data Availability

The dataset generated and analysed in this study is available as supplementary information.
