# Supplementary material for: Cross-modal size-contrast illusion: Acoustic increases in intensity and bandwidth modulate haptic representation of object size
Source: Sci Rep. 2019 Oct 8;9:14440. doi: 10.1038/s41598-019-50912-8 (PMC6783429; doi:10.1038/s41598-019-50912-8)
Supplement: Supplementary file 1 — Dataset 1 [file 41598_2019_50912_MOESM1_ESM.pdf]

Cross-modal size-contrast illusion: Acoustic increases in intensity and bandwidth modulate haptic representation of object size

Authors:
Maiko Uesaki[*1,2,3], Hiroshi Ashida[4], Akiyoshi Kitaoka[2,5] and Achille Pasqualotto[1,6,7]

Affiliations:
1. School of Social Sciences, Nanyang Technological University, Singapore
2. Open Innovation & Collaboration Research Organization, Ritsumeikan University, Osaka, Japan
3. Japan Society for the Promotion of Science, Tokyo, Japan
4. Graduate School of Letters, Kyoto University, Kyoto, Japan
5. College of Letters, Ritsumeikan University, Kyoto, Japan
6. Faculty of Arts and Social Sciences, Sabanci University, Istanbul, Turkey
7. School of Psychology, Faculty of Science and Engineering, University of Nottingham Malaysia, Selangor Darul Ehsan, Malaysia

Corresponding author:
Maiko Uesaki
School of Social Sciences, Nanyang Technological University, Singapore
maiko.uesaki@ntu.edu.sg

Forward Condition

Participant	Probe	Estimation	Error	Average error
S1	15	10	-5	-5.333333333
	25	21	-4	
	40	33	-7	
S2	15	7	-8	-6.666666667
	25	20	-5	
	40	33	-7	
S3	15	10	-5	-6.666666667
	25	20	-5	
	40	30	-10	
S4	15	10	-5	-6.666666667
	25	20	-5	
	40	30	-10	
S5	15	20	5	5
	25	25	0	
	40	50	10	
S6	15	11	-4	-8.666666667
	25	17	-8	
	40	26	-14	
S7	15	15	0	1.666666667
	25	30	5	
	40	40	0	
S8	15	10	-5	-1.666666667
	25	25	0	
	40	40	0	
S9	15	7	-8	-11
	25	15	-10	
	40	25	-15	
S10	15	10	-5	-8.333333333
	25	15	-10	
	40	30	-10	
S11	15	10	-5	-6.666666667
	25	20	-5	
	40	30	-10	
S12	15	12	-3	-6
	25	20	-5	
	40	30	-10	
S13	15	5	-10	-14.66666667
	25	11	-14	
	40	20	-20	
S14	15	12	-3	-6.666666667
	25	18	-7	
	40	30	-10	
S15	15	18	3	-2.333333333
	25	25	0	
	40	30	-10	
S16	15	10	-5	-3.333333333
	25	25	0	
	40	35	-5	
S17	15	9	-6	-5.333333333
	25	20	-5	
	40	35	-5	
S18	15	10	-5	-3.333333333
	25	25	0	
	40	35	-5	
S19	15	16	1	-1.333333333
	25	25	0	
	40	35	-5	
S20	15	15	0	1.666666667
	25	30	5	
	40	40	0	
S21	15	11	-4	-6.333333333
	25	18	-7	
	40	32	-8	
S22	15	20	5	0
	25	20	-5	
	40	40	0	

Backward Condition

Participant	Probe	Estimation	Error	Average error
S23	15	20	5	2.666666667
	25	28	3	
	40	40	0	
S24	15	20	5	5
	25	25	0	
	40	50	10	
S25	15	20	5	3.333333333
	25	25	0	
	40	45	5	
S26	15	10	-5	-5
	25	20	-5	
	40	35	-5	
S27	15	9	-6	-3.666666667
	25	22	-3	
	40	38	-2	
S28	15	15	0	-3.333333333
	25	20	-5	
	40	35	-5	
S29	15	12	-3	4
	25	30	5	
	40	50	10	
S30	15	20	5	5
	25	30	5	
	40	45	5	
S31	15	15	0	-0.666666667
	25	25	0	
	40	38	-2	
S32	15	12	-3	-4.333333333
	25	20	-5	
	40	35	-5	
S33	15	15	0	-3.333333333
	25	20	-5	
	40	35	-5	
S34	15	15.24	0.24	-0.42
	25	25.4	0.4	
	40	38.1	-1.9	
S35	15	20	5	6.666666667
	25	30	5	
	40	50	10	
S36	15	12	-3	-2.333333333
	25	25	0	
	40	36	-4	
S37	15	10	-5	-1.666666667
	25	30	5	
	40	35	-5	
S38	15	15	0	11.66666667
	25	40	15	
	40	60	20	
S39	15	10	-5	-6.666666667
	25	20	-5	
	40	30	-10	
S40	15	10	-5	0
	25	20	-5	
	40	50	10	
S41	15	15	0	5
	25	30	5	
	40	50	10	
S42	15	20	5	6.666666667
	25	30	5	
	40	50	10	
S43	15	10	-5	-6
	25	20	-5	
	40	32	-8	
S44	15	15	0	-3.333333333
	25	25	0	
	40	30	-10	

Scrambled Condition

Participant	Probe	Estimation	Error	Average error
S45	15	20	5	5
	25	30	5	
	40	45	5	
S46	15	10	-5	-4.666666667
	25	21	-4	
	40	35	-5	
S47	15	8	-7	-12.33333333
	25	15	-10	
	40	20	-20	
S48	15	8	-7	-7.333333333
	25	20	-5	
	40	30	-10	
S49	15	10	-5	-6.666666667
	25	20	-5	
	40	30	-10	
S50	15	15	0	0
	25	25	0	
	40	40	0	
S51	15	14	-1	-4.333333333
	25	18	-7	
	40	35	-5	
S52	15	20	5	0.666666667
	25	27	2	
	40	35	-5	
S53	15	10	-5	1
	25	23	-2	
	40	50	10	
S54	15	10	-5	1.666666667
	25	25	0	
	40	50	10	
S55	15	10	-5	-6.666666667
	25	20	-5	
	40	30	-10	
S56	15	13	-2	-6.333333333
	25	18	-7	
	40	30	-10	
S57	15	15	0	-5
	25	20	-5	
	40	30	-10	
S58	15	15	0	8.333333333
	25	40	15	
	40	50	10	
S59	15	25	10	0
	25	20	-5	
	40	35	-5	
S60	15	12	-3	2.333333333
	25	30	5	
	40	45	5	
S61	15	15	0	-0.666666667
	25	23	-2	
	40	40	0	
S62	15	12	-3	-2.333333333
	25	26	1	
	40	35	-5	
S63	15	12	-3	-5
	25	23	-2	
	40	30	-10	
S64	15	15	0	2.666666667
	25	23	-2	
	40	50	10	
S65	15	22	7	17.33333333
	25	50	25	
	40	60	20	
S66	15	10	-5	6.666666667
	25	35	10	
	40	55	15	
